# Lipid-based nanocarrier efficiently delivers highly water soluble drug across the blood–brain barrier into brain

**DOI:** 10.1080/10717544.2018.1435749

**Published:** 2018-02-09

**Authors:** Lopamudra Dutta, Biswajit Mukherjee, Tapash Chakraborty, Malay Kumar Das, Laboni Mondal, Sanchari Bhattacharya, Raghuvir H. Gaonkar, Mita Chatterjee Debnath

**Affiliations:** a Department of Pharmaceutical Technology, Jadavpur University, Kolkata, India;; b Department of Pharmaceutical Sciences, Dibrugarh University, Dibrugarh, India;; c Infectious Diseases and Immunology Division, CSIR-Indian Institute of Chemical Biology, Kolkata, India

**Keywords:** Nanosize lipid carrier, blood–brain barrier, gamma scintigraphy, pharmacokinetics, biodistribution

## Abstract

Delivering highly water soluble drugs across blood–brain barrier (BBB) is a crucial challenge for the formulation scientists. A successful therapeutic intervention by developing a suitable drug delivery system may revolutionize treatment across BBB. Efforts were given here to unravel the capability of a newly developed fatty acid combination (stearic acid:oleic acid:palmitic acid = 8.08:4.13:1) (ML) as fundamental component of nanocarrier to deliver highly water soluble zidovudine (AZT) as a model drug into brain across BBB. A comparison was made with an experimentally developed standard phospholipid-based nanocarrier containing AZT. Both the formulations had nanosize spherical unilamellar vesicular structure with highly negative zeta potential along with sustained drug release profiles. Gamma scintigraphic images showed both the radiolabeled formulations successfully crossed BBB, but longer retention in brain was observed for ML-based formulation (MGF) as compared to soya lecithin (SL)-based drug carrier (SYF). Plasma and brain pharmacokinetic data showed less clearance, prolonged residence time, more bioavailability and sustained release of AZT from MGF in rats compared to those data of the rats treated with SYF/AZT suspension. Thus, ML may be utilized to successfully develop drug nanocarrier to deliver drug into brain across BBB, in a sustained manner for a prolong period of time and may provide an effective therapeutic strategy for many diseases of brain. Further, many anti-HIV drugs cannot cross BBB sufficiently. Hence, the developed formulation may be a suitable option to carry those drugs into brain for better therapeutic management of HIV.

## Introduction

Blood–brain barrier (BBB), a complex tight endothelial vascular lining, is the main hindrance of most chemicals for free diffusion and penetration into the brain from blood stream of body for maintaining homeostasis in brain (Seju et al., 2011; Martins et al., 2012). BBB acts as a safeguard of brain from exogenous toxic agents as well as rejecter of essential therapeutic agents (Hu et al., 2009). Nearly 100% of large molecular drugs and about 98% of drugs consisting of small molecules are unable to cross BBB to provide therapeutic outcome (Wilson et al., 2008; Hu et al., 2017). Various novel drug delivery systems such as nanoparticles, nanoliposomes (NLs), micelles, dendrimers, quantum dots, and nanoemulsions are applied to overcome the limitations (Li et al., 2017). Nowadays, nanosize drug delivery into brain across BBB is an emerging field of pharmaceutical research. In the current study, we have selected lipid-based nanoliposomal drug carrier to deliver drug into brain. NLs can deliver hydrophobic as well as hydrophilic drug efficiently due to their special structure. Due to some important properties such as biodegradability, biocompatibility, low toxicity, ability of enhancement of therapeutic index and efficacy of drug, enhancement of stability of drug through encapsulation, and non-immunogenicity, the liposomal drug delivery is a choice as a drug carrier (Akbarzadeh et al., 2013).

**Figure 1.**
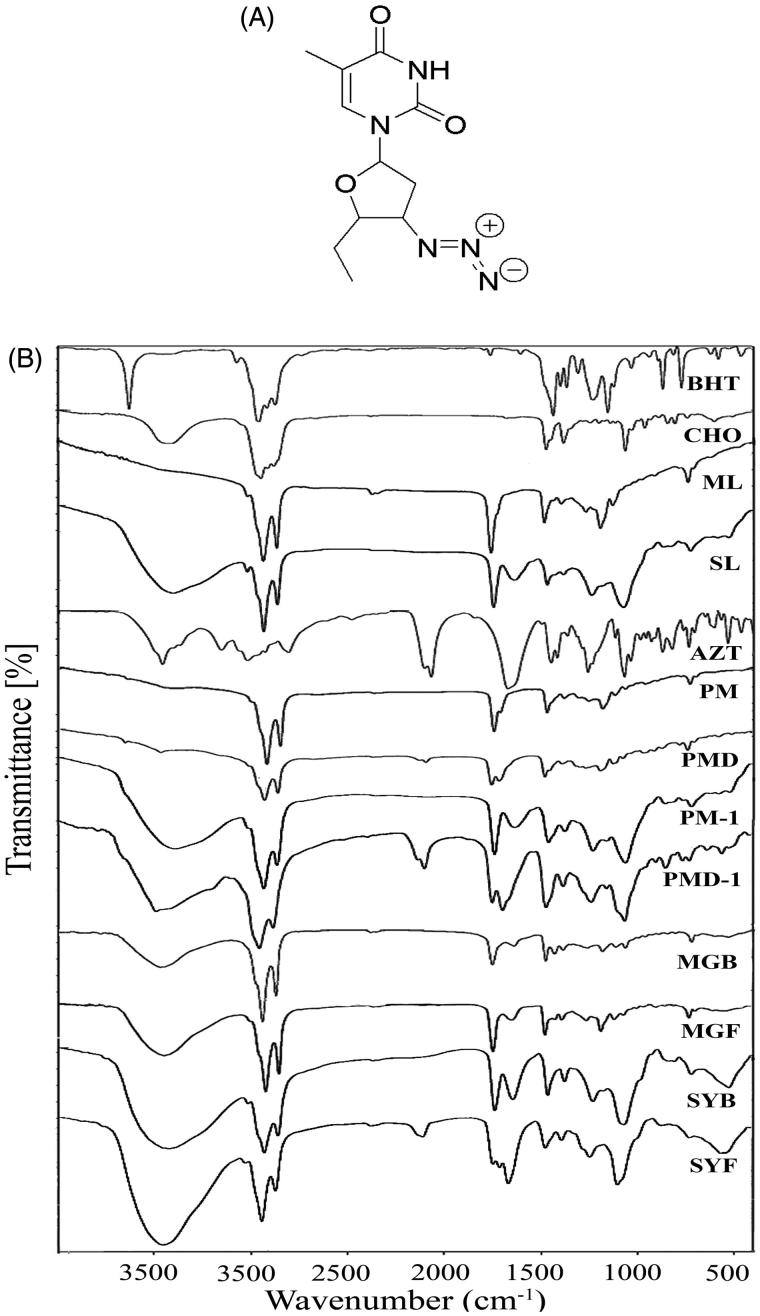
(A) Chemical structure of zidovudine (AZT). (B) Fourier transform infrared spectroscopy (FTIR)-spectra of BHT (butylated hydroxytoluene), CHO (cholesterol), ML (lipid), SL (soya lecithin), AZT (zidovudine), PM (physical mixture of BHT, CHO, ML), PMD (physical mixture of BHT, CHO, ML, and drug), PM-1 (physical mixture of BHT, CHO, SL), PMD-1 (physical mixture of BHT, CHO, SL, and drug), MGB (ML-based lyophilized formulation without drug), MGF (ML-based lyophilized formulation with drug), SYB (SL-based lyophilized formulation without drug), and SYF (SL-based lyophilized formulation with drug).

It is always an enormous challenge for formulation scientists to deliver highly water soluble drug into brain across BBB and the same is true for many other large molecules. In the present study, we have selected a highly water soluble drug zidovudine (AZT), 1-[(2R,4S,5S)-4-azido-5-(hydroxymethyl)oxolan-2-yl]-5-methylpyrimidine-2,4-dione (Figure 1(A)), as a model water-soluble drug to deliver across BBB into brain. AZT is a highly water soluble drug (25 mg/ml at 25 °C). Therefore, it has been used as a representative water soluble active pharmaceutical ingredient or drug in a number of reports (Jain et al., 2008; Nayak et al., 2009; Singh et al., 2010; Christoper et al., 2014). It is expected that the physical characteristics provided by AZT would be similar for many other hydrophilic drugs. Hence, we have considered AZT as a water soluble model drug in the present study. AZT, nucleoside reverse transcriptase inhibitor, is also a part of combination therapy ‘highly active antiretroviral treatment (HAART)’ for anti-HIV treatment (Bergshoeff et al., 2004; Mu et al., 2016). AZT due to its strong hydrophilicity is unable to cross BBB sufficiently to reach brain, resulting in inadequate concentration of AZT for therapeutic efficacy (Rautio et al., 2008; Weiss et al., 2009). Thus, delivering AZT in brain could be utilized for better management of HIV as in HIV infected patients, at very early stage (up to 10 days of post HIV infection), neuro-invasion can arise and HIV infected circulated monocytes in blood stream can easily enter into brain (Ivey et al., 2009).

Here, by trial and error method we developed several lipids using various mixtures of three fatty acids present in many edible lipids (Kittiphoom, 2012). Out of them we have selected the best combination [stearic acid (SA):oleic acid (OA):palmitic acid (PA) = 8.08:4.13:1] (ML) on the basis of its consistency to develop the nanocarrier. We also compared the efficacy of this formulation with soya lecithin (SL)-based formulation developed by us.

The prime objective of this investigation was to evaluate the capability of ML as base component for the development of nanocarrier to deliver highly water soluble drug into brain across BBB. Further, its efficiency was evaluated by comparing with SL-based drug nanocarrier.

## Materials and methods

AZT was obtained as a gift sample from Cipla Ltd. (Goa, India). Cholesterol (CHO), fluorescein isothiocyanate (Isomer I) (FITC), SL, fetal bovine serum (FBS) and minimum essential medium Eagle (MEM) were procured from HiMedia Laboratories Pvt. Ltd. (Mumbai, India). SA, OA, and PA were purchased from Sigma-Aldrich (Bangalore, India) and butylated hydroxytoluene (BHT) was obtained from Qualigens Fine Chemicals (Mumbai, India). U-87 MG cells were procured from National Center for Cell Science (Pune, India). All other chemicals used were of analytical grade.

### Animals

Sprague-Dawley rats (male:female ratio 2:1) having body weight 200–250 g were utilized for biodistribution investigation, plasma and brain pharmacokinetic study and only male Sprague-Dawley rats of body weight 200–250 g were used for gamma scintigraphy study. Animal experiments were carried out upon receiving approval of the Animal Ethics Committee, Jadavpur University, Kolkata. Animals were accommodated in the university animal house after keeping them in polypropylene cages. The temperature (22 ± 1 °C) and humidity (55 ± 5%) were maintained in the animal house with 12 h light/dark circle. The animals had free access of standard diet (Dey et al., 2016) and drinking water.

### Fourier-transform infrared spectroscopy (FTIR)

FTIR was conducted to determine possible interaction (if any) between the drug and the excipients. For ML-based formulation, pure drug (AZT), CHO, ML, BHT, physical mixture (PM) of CHO, ML, BHT, physical mixture (PMD) of CHO, ML, BHT and drug AZT, lyophilized formulation without drug (MGB) and lyophilized formulation with drug (MGF) and for SL-based formulation, AZT, CHO, SL, BHT, physical mixture (PM-1) of CHO, SL, BHT, physical mixture (PMD-1) of CHO, SL, BHT and AZT, lyophilized formulation without drug (SYB) and lyophilized formulation with drug (SYF) were scanned at 4000–400 cm^−1^ using FTIR instrument (JASCO International Co. Ltd., FTIR 4200, Tokyo, Japan) using their pellets formed by mixing with potassium bromide (KBr) at 1:100 ratio and compressing with a hydraulic press (Sahana et al., 2010).

### Preparation of NLs

Fatty acids at the selected ratio were dissolved in small quantity of chloroform and chloroform was evaporated under vacuum to get the lipid formed from the fatty acids.

NLs were prepared by lipid layer hydration technique by varying different process parameters (Rudra et al., 2010). Specific weighed amounts of excipients CHO, lipids (ML for MGF/SL for SYF, respectively) (Supplementary Table 1) and BHT (1% w/v as antioxidant) were taken in 250 ml round bottom flask and adequate quantity of chloroform was added within the flask with vigorous shaking to dissolve the excipients. The flask was set up in a rotary vacuum evaporator (Rotavap, model PBU-6, Superfit Continental Pvt. Ltd., Mumbai, India) fitted with an A3S aspirator (Eyela, Rikakikaic, Ltd., Taguig City, Philippines) and a circulating water bath (Spac N service, Kolkata, India) at 5 °C and was rotated at 145 rpm rotation speed at 40 °C in water bath to evaporate the organic solvent and to form thin film of lipid layer on the inside-wall of the flask. For complete elimination of the residual chloroform, the flask was kept overnight in a vacuum desiccator. The weighed amount of AZT was dissolved in phosphate buffer (pH 7.4), and the mixture was taken in the flask. The flask was then fitted in a rotary vacuum evaporator with rotation speed at 145 rpm and at 40 °C in a water-bath for complete dispersion of thin lipid film in the aqueous phase. The dispersion was sonicated at 30 ± 3 kHz in a bath type sonicator (Trans-o-Sonic, Mumbai, India) for 1 h with cold water. To form vesicles, the round bottom flask was kept at room temperature for 1.5 h and stored overnight at 4 °C. The preparation was centrifuged at 5000 rpm for 15 min at 4 °C to separate the larger vesicles and the obtained supernatant was again centrifuged (at 16,000 rpm for 45 min in a cold centrifuge at 4 °C) (3K30 Sigma Lab Centrifuge, Merrington Hall Farm, Shrewsbury, UK) to precipitate NLs. The obtained precipitate was re-suspended in fresh phosphate buffer (pH 7.4) and precipitated again number of times to wash the NLs. NLs were collected in a petridish and lyophilized for 12 h (Laboratory Freeze Dryer, Instrumentation India Ltd., Kolkata, India) to get dry product. Blank NLs (MGB, SYB) were prepared by the same procedure without using AZT. Fluorescent NLs were prepared using the same process as described above except the step where fluorescent marker FITC was used. FITC stock solution (0.4% w/v) was prepared in a mixture of chloroform and ethanol at volume 3:1 ratio and a volume of 100 µl was mixed into the organic phase (chloroform) during the initial mixing of preparation (Shaw et al., 2017).

### Characterization of NL

#### Evaluation of drug loading

MGF/SYF (5 mg) was taken in a mixture of phosphate buffer (pH 7.4) and ethanol at a ratio of 5:1 and sonicated and vortex-mixed. After centrifugation (at 16,000 rpm for 15 min at 4 °C), the supernatant was collected and measured at 265 nm using UV/VIS spectrophotometer (Model Intech-295, Gentaur GmbH, Aachen, Germany). The same procedure was performed for MGB/SYB. Absorbance for drug was obtained by deducting the absorbance obtained from NLs without drug and from that of NLs with drug (Satapathy et al., 2016). Each study was conducted thrice. The percentage of drug loading was calculated using the following formula:

(1) Percentage of drug loading = (Amount of AZT in NLs/Amount of NLs taken) * 100

#### Percentage of yield determination

To determine the yield of NLs from the utilized total amount of raw materials of NLs preparation, lyophilized dried NLs of each batch were weighed and the percentage of yield was calculated by using the following equation as mentioned earlier (Sahana et al., 2010).

(2) Percentage of yield = (Amount of NLs obtained/Total amount of drug and excipients used) * 100

#### Evaluation of size distribution and zeta potential

By utilizing the dynamic light scattering (DLS) technology in a Zetasizer Nano ZS90 (Malvern Instrument, Malvern, UK), average size of NLs, size distribution pattern, polydispersity index (PDI) and zeta potential of the experimental formulations were determined.

#### Field emission scanning electron microscopy (FESEM) for surface morphology analysis

FESEM was used to investigate the surface morphology of NLs. The lyophilized NLs were placed on an adhesive tape of carbon over a stub by spreading smoothly and then dried through vacuum and coated with platinum using a platinum coater instrument (JEOL, Tokyo, Japan). After that the samples were observed at various magnifications with the help of FESEM (Model-JSM-6700F; JEOL, Tokyo, Japan) (Dey et al., 2016).

#### Cryo-transmission electron microscopy (Cryo-TEM) study

Cryo-TEM study was performed to observe morphology and lamellarity of NLs. About 1.5 mg lyophilized formulation was taken in a microcentrifuge tube and 1 ml of Milli-Q water was added into it. The suspension was vortexed followed by sonication for a few minutes to prevent agglomeration. The NLs suspension (4 µl) was put on a clean grid, blotted away the excess (if any) with the help of filter paper and vitrified instantly by dipping into liquid ethane. The grid was stored in liquid nitrogen till shifting under the electron microscope (Tecnai Polora, version 4.6 FEI Tecnai G2, Eindhoven, Netherlands) which was operated at 300 kV equipped with an FEI Eagle 4 K × 4 K charge-coupled device (CCD) camera for capturing the images (Fox et al., 2014).

### *In vitro* investigations

#### *In vitro* drug release study

*In vitro* drug release study was conducted in freshly prepared phosphate buffer (pH 7.4) and in 50% serum [serum:phosphate buffer (pH 7.4) = 1:1] individually as drug release media at room temperature (for phosphate buffer as media) and at 37 °C (for 50% serum as media) (in physiological mimicking condition), for 24 h. Drug release media (50 ml) were taken in a glass beaker (100 ml). Lyophilized formulation (5 mg) was weighed accurately and reconstituted in 1 ml of the respective drug release media and was placed into a dialysis bag (Himedia Dialysis Membrane – 110, Mumbai, India) (Dey et al., 2016). The dialysis bag was tightly knotted at the two ends with cotton thread and hanged centrally into the beaker containing drug release medium (in such a way so that the formulation portion inside the bag immersed within the media) using a laboratory ring stand with clamp kit. The beaker was put on a magnetic stirrer maintaining stirring at 300 rpm using a magnetic bead. From the beaker, 1 ml media was withdrawn at various predetermined time intervals and same volume of fresh media was replaced immediately into the beaker. All the collected samples were analyzed using UV-VIS spectrophotometer at 265 nm with phosphate buffer or 50% serum as blank according to requirement. In case of serum sample, protein was precipitated as mentioned under LC-MS/MS study. The drug concentration at any individual time point was calculated with the help of calibration curve. For 50% serum as drug release media, total experiment was conducted maintaining aseptic condition in all respect under laminar air flow hood.

#### *In vitro* drug release kinetic study

*In vitro* drug release data were applied in various kinetic models such as zero order (percentage cumulative drug release versus time), first order (log of percentage cumulative amount of drug remained to release versus time), Higuchi model (percentage cumulative drug release versus square root of time), Hixson–Crowell’s model (cube root of percentage cumulative amount of drug remained to release versus time), Korsmeyer–Peppas’s model (log of percentage cumulative drug release versus log of time) to predict the drug release pattern of the optimized formulations. The highest correlation coefficient value (*R*^2^) from all the tested models was utilized to select the suitable kinetic pattern (Das et al., 2015).

#### *In vitro* cellular uptake study

To investigate the cellular uptake ability of NLs by U-87 MG cells, confocal laser scanning microscopy study was executed. In six well culture plates, U-87 MG cells were seeded on coverslips (3 × 10^4^) and cultivated using MEM containing 10% FBS for 24 h. These cells were treated with FITC-MGF/FITC-SYF suspension at a concentration of 100 μg/ml. At different time points (i.e. 0.25 h, 1 h, 3 h) of incubation, the cells were washed thrice and fixed applying paraformaldehyde aqueous solution (4%). They were cleaned by using freshly prepared phosphate buffer (pH 7.4) after 5 min of fixation. Then, coverslips were collected cautiously and mounted on the glass slide. After complete air drying, the slides were placed individually under a confocal laser scanning microscope (Model: IX81, Olympus Singapore Pte Ltd., Singapore) and the images were snapped (Maji et al., 2014).

### Stability study

Stability testing of the lyophilized formulations was performed as per ICH guidelines (Sahana et al., 2010).

### *In vivo* investigations

#### Radiolabeling of AZT and AZT loaded NLs

According to the tin (II) chloride reduction method as described earlier (Das et al., 2015; Satapathy et al., 2016), radiolabeling of AZT and AZT loaded MGF/SYF was performed with technetium-99m (^99m^Tc). At first, 5 mg of AZT was dissolved in 0.5 ml ethanol and AZT loaded NLs (equivalent to 5 mg of AZT) were suspended in 0.5 ml of nitrogen purged water. The aqueous ^99m^Tc-pertechnetate (^99m^TcO4^–^) (40–100 MBq) was incorporated to them followed by addition of 25 μl of aqueous stannous chloride dihydrate (SnCl_2_·2H_2_O) (2 mg/ml) solution. At room temperature, they were incubated for 15 min. The radiolabeled efficiencies were then assessed with the help of ascending thin layer chromatography by applying silica gel coated aluminum strips (Merck, Darmstadt, Germany) as stationary phase and methyl ethyl ketone as mobile phase. The sheets were dried after developing the spots and they were cut into five strips (1 cm each). These were analyzed quantitatively through counting using a well type gamma counter at 140 keV (Electronic Corporation of India, model LV4755, Hyderabad, India).

#### Gamma scintigraphy

For providing direct evidence of location of radiolabeled NLs as well as free drug within the body of experimental rats, gamma scintigraphy imaging was performed. Only male Sprague-Dawley rats (body weight 200–250 g) were utilized in this study. After dividing the total rats into three groups, ^99m^Tc labeled AZT/^99m^Tc labeled MGF/^99m^Tc labeled SYF was injected (100 μl) through femoral vein of rats of the respective group. Using the intramuscular injection of ketamine hydrochloride (1 ml), rats were anesthetized and fixed on a board in the posterior position for imaging. At predetermined time interval (1 h and 5 h of post injection), static images were snapped with the help of planar gamma camera (GE Infinia Gamma Camera equipped with Xeleris Workstation, GE, Cleveland, OH).

#### Biodistribution study

Sprague-Dawley rats (body weight 200–250 g) were utilized for performing the biodistribution of radiolabeled AZT and NLs (MGF, SYF). By applying ketamine (30–50 mg/kg) intramuscularly, rats were anesthetized and cannulation was done in the femoral vein of animals using polyethylene (PE-50) catheter tubes. All the experimental animals were well-hydrated by administering (2 ml) normal saline (0.9% NaCl (w/v) in water) through intraperitoneal route for 1 h. ^99m^Tc labeled AZT/^99m^Tc labeled MGF/^99m^Tc labeled SYF was injected at 0.03 ml volume (10–15 MBq/kg) via intravenous (i.v.) route through the cannula. The animals were sacrificed at 1 h and 5 h post injection. The organs and tissues such as heart, liver, lung, spleen, muscle, intestine, stomach, kidney, and brain were removed followed by washing using normal saline. The collected organs and tissues were dried up immediately using blotting paper (if applicable) and taken into the preweighed counting vials. Blood was collected using heart puncture process. With the help of a well-type gamma scintillation counter along with an injection standard, the corresponding radioactivity of the samples was measured and percentage of injected dose per gram (% ID per g) of tissue or organ was utilized for expressing the results.

#### *In vivo* plasma and brain pharmacokinetic study

*In vivo* plasma and brain pharmacokinetic study was performed to compare the distribution of AZT from free drug suspension and NLs (MGF, SYF) and observe the ability of NLs to cross BBB in Sprague-Dawley rats. The animals were distributed into four groups. AZT suspension was injected in animals of one group through i.v. route as per dose. Another group of animals was injected MGF and the third group was treated with SYF intravenously with an equivalent amount of AZT with respect to free drug in suspension. Tail vein was selected to administer the injection for all animals of each batch. The fourth group remained as control (untreated). For plasma pharmacokinetic study, blood samples were collected by terminal cardiac puncture of each animal following anesthesia, at a predetermined time interval such as 0.25 h, 0.5 h, 1 h, 2 h, 4 h, 6 h, 8 h, 10 h, 12 h, 24 h, and 48 h of post i.v. injection and kept the sample immediately into a microcentrifuge tube having EDTA solution. For plasma collection, the blood samples were centrifuged at 5000 rpm for 6 min using cold centrifuge (HERMLE Labortechnik GmbH, Wehingen, Germany) and the plasma was preserved at –80 °C till further analysis (Dey et al., 2016).

For brain kinetic study, at a predetermined time interval (0.5 h, 1 h, 2 h, 4 h, 6 h, 8 h, 10 h, 12 h, and 24 h of post i.v. injection), rats were dissected and brains were separated followed by washing with Milli-Q water. *In situ* blood perfusion (Takasato et al., 1984) was done before collection of brain in the experiment. Then, the collected brains were kept into cryogenic tubes and stored at –80 °C till LC–MS/MS study.

#### LC–MS/MS study

Evaluation of AZT concentration in plasma and brain was performed by using LC–MS/MS technique (Shaw et al., 2017). Briefly, plasma sample/homogenized brain tissue was first mixed with ice cold acetonitrile (plasma/tissue homogenate:cold acetonitrile was 1:3 by volume) containing internal standard followed by vortex-mixed for 10 min and centrifuged (at 4000 rpm for 15 min at 4 °C) for efficient extraction of AZT by protein precipitation method (Gautam et al., 2013). The supernatant for each sample was collected. The supernatant (100 µl) of each sample was mixed with 100 µl water and loaded into LC–MS/MS (LC: Shimadzu Model 20AC, MS: AB-SCIEX, Model: API4000, Software: Analyst 1.6). Elution was done with the help of YMC Triat C18 column (2.1 × 30 mm, 5 µ). Gradient elution technique of two mobile phases (mobile phase A: 0.1% formic acid in water and mobile phase B: 0.1% formic acid in 80:20 acetonitrile/water) was conducted with injection volume: 20 µl, flow rate 0.8 ml/min and total run time was 3.0 min in each case.

#### Calculation of PK parameter

AZT concentrations in plasma and brain were plotted against time. By utilizing NCA toolbox of Phoenix-Winnonlin software (Certara, Daresbury, UK), various PK parameters such as area under the concentration–time curve from time of injection to a determined time point (AUC_0–_*_t_*), area under the first moment curve (AUMC_0–_*_t_*), clearance (Cl), time taken for maximum blood concentration to drop in half-life (*t*_1/2_), steady state volume of distribution (*V*_ss_), mean residence time (MRT), etc. were determined.

#### Statistical analysis

Statistical calculations of various data were conducted by applying one-way analysis of variance (ANOVA) through Tukey–Kramer’s multiple comparisons test with the help of GraphPad InStat (version 3.06) software (La Jolla, CA). The probability value (*p*) <.05 at 95% confidence interval was recognized as statistically significant.

## Results

### FTIR analysis

Drug–excipient interaction was investigated through FTIR spectroscopy to determine any interaction between the functional groups of drug (AZT) and the excipients of a formulation (Basu et al., 2012). When the FTIR spectra of the physical mixture of the drug and the excipients (ML, CHO) were compared with those of the physical mixture of the excipients without drug, the lyophilized formulation with or without drug and each of the excipients, the presence of the characteristic peaks of the drug (at 2083 cm^−1^), ML (at 2922 cm^−1^, at 2852 cm^−1^, and at 721 cm^−1^) and CHO (at 1373 cm^−1^) was observed (Figure 1(B)).

Likewise, during the comparison of the FTIR spectra of the drug, SL and CHO, the presence of the characteristic peak of the drug was observed at 2083 cm^−1^ in the physical mixture. The characteristic peaks of SL (at 2928 cm^−1^ and at 1462 cm^−1^) and CHO (at 1373 cm^−1^) were also observed in the physical mixture of the drug and the excipients (Figure 1(B)).

The findings suggest the absence of any chemical interaction between the drug and the excipients for both the formulations (ML based, MGF and SL based, SYF). However, minor shifting of some of the peaks of the excipients in formulations (from 2922 cm^−1^ to 2920 cm^−1^, 2852 cm^−1^ to 2851 cm^−1^, and from 721 cm^−1^ to 719 cm^−1^ in ML-based formulation and CHO from 3415 cm^−1^ to 3436 cm^−1^ in case of SL-based formulation) was observed. The observations suggest that physical interactions existed between the molecules of the excipients. The interaction might be responsible to provide the structure of the formulation and for sustained drug release from the formulations. In case of the ML formulation, the peak of the drug was not observed. The finding suggests that the drug was encapsulated entirely in the formulation and there was the absence of drug molecules on the surface of the formulation. For SL formulation, presence of the peak of the drug was found to shift from 2084 cm^−1^ to 2096 cm^−1^. The finding indicates that the shifting of the peak of the drug might be due to physical interaction between the drug and the excipients. Further, the drug molecules were also present in the lipid bilayers and might retain in the formulation due to the physical interaction between the drug and the excipients.

In case of SL formulation, a shifting of wave number from 2084 cm^−1^ to 2096 cm^−1^ might be due to the formation of weak bond (such as hydrogen bond, van der Waals force of attraction or dipole moment) between methyl group of the drug and OH group of CHO. The wave number ranging from 3415 cm^−1^ to 3436 cm^−1^ is the strong intensity stretching vibration zone of OH group. The wave number ranging from 2084 cm^−1^ to 2096 cm^−1^ is the stretching vibration zone of C–C=C of the drug. Further, the wave number regions, 2922–2920 cm^−1^ and 2852–2851 cm^−1^, are the strong intensity stretching vibration regions of CH_2_, CH, and CH_3_ and the region 721–719 cm^−1^ is the variable to weak intensity bending vibration region of out of plane OH bending. Hence, there might be physical interaction by formation of weak bonds (hydrogen bond, van der Waals force of attraction or dipole moment) responsible for minor shifting of peaks.

### Physico-chemical characterization of NLs

We initially varied the different process parameters such as temperature and speed of hydration, duration of sonication, time and speed of centrifugation, duration of freeze drying and ratio of the constituents to optimize the preparation process and to select the formulations. The best two formulations (MGF, SYF) were selected based on drug loading, particle size, zeta potential, FESEM, and Cryo-TEM data and investigated for further study.

#### Percentage of drug loading and yield

The percentage of drug loading of MGF was 5.7 ± 0.37% with 60.87 ± 5.92% yield capacity but SYF had 7.00 ± 0.23% drug loading with yield capacity of 54.34 ± 4.79% (Supplementary Table 1). Every experiment was repeated thrice to establish the reproducibility.

#### Size, size distribution, and zeta potential

**Figure 2.**
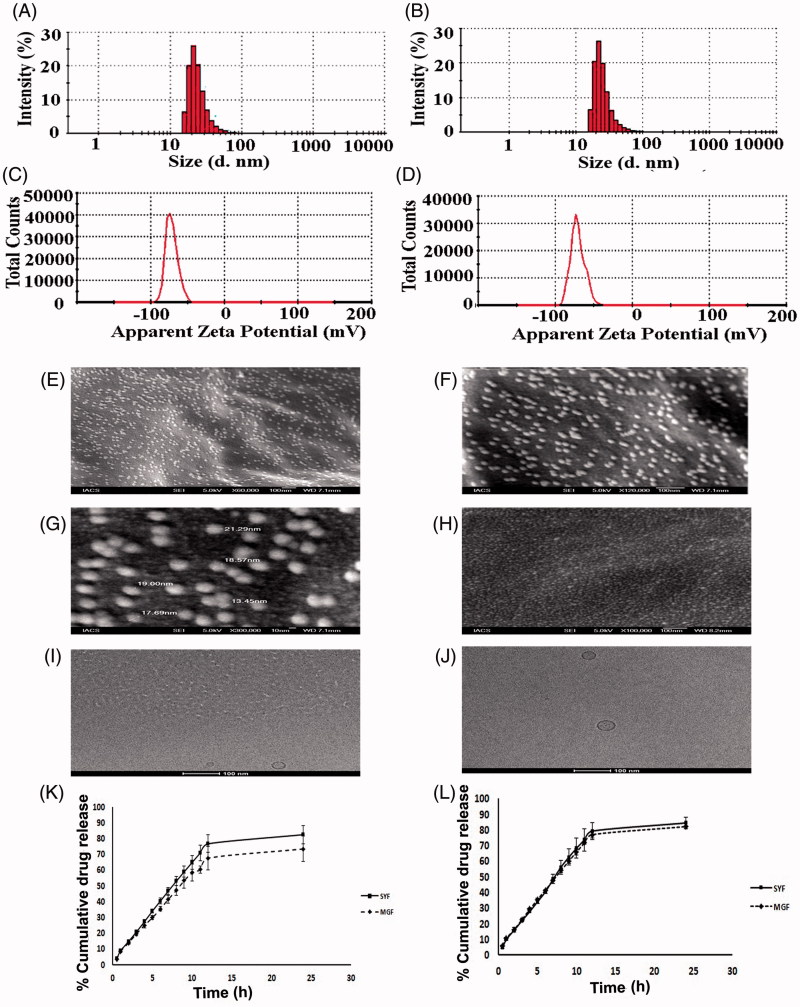
Particle size distribution of (A) MGF and (B) SYF. Zeta potential of (C) MGF and (D) SYF. Field emission scanning electron microscopy (FESEM) images of (E) MGF at magnification of 60,000×, (F) MGF at magnification of 120,000×, (G) MGF at magnification of 300,000×, (H) SYF at magnification of 100,000×. Cryo-transmission electron microscopy (Cryo-TEM) images of (I) MGF and (J) SYF. Scale bar for Cryo-TEM image: 100 nm. (K) *In vitro* drug release profiles of MGF and SYF in phosphate buffer (pH 7.4). (L) *In vitro* drug release profiles of MGF and SYF in 50% serum. Data show mean ± SD (*n* = 3). SD: standard deviation.

Liposomal size, surface morphology, and the lamellarity of liposome have been assessed by particle size analyzer, FESEM, and Cryo-TEM, respectively. FESEM figures showed that the average size of the lyophilized liposome varied for MGF and SYF. When the liposomes were assessed by particle size analyzer, it showed the average size 24.37 ± 2.2 nm for MGF (Figure 2(A)) and 33.75 ± 3.7 nm for SYF (Figure 2(B)), with their PDI 0.26 ± 0.02 and 0.25 ± 0.06, respectively. When the lamellarity was checked by Cryo-TEM, average size of the vesicles was found to be 30.75 ± 1.8 nm for MGF and 40.43 ± 2.4 nm for SYF with the intact lamellarity. The size was little bit higher as compared to the lyophilized liposome and the liposome analyzed by particle size analyzer. Since for Cryo-TEM analysis, the lyophilized liposomes were suspended for longer time as compared to particle size analysis by particle size analyzer, the average size was enhanced marginally due to the entrapment of water in the core area of the liposomal vesicles. However, all the methods of analysis showed that the size was below 50 nm. Zeta potential of both the formulations was observed as negative, –71.5 ± 6.5 mV (Figure 2(C)) and –70.8 ± 7.0 mV (Figure 2(D)) for MGF and SYF, respectively. The statistical analysis of data of average size for both the formulations and the variation in PDI showed that there was an insignificant variation (*p* > .05) in size distribution, but a significant variation of particle size between MGF and SYF. MGF showed smaller size particles compared to SYF. Zeta potential showed nearly same value for both the formulations.

#### FESEM study

Three images of FESEM at different magnifications namely 60,000×, 120,000×, and 300,000× were captured for MGF to establish the shape, size, and distribution pattern of the formulation. The MGF had nanosize structure with smooth surface and was homogenously distributed at 60,000× and 120,000× magnification (Figure 2(E,F)). At 300,000× magnification, MGF was found to be spherical structure having nanosize with smooth surface and a homogeneous distribution pattern (Figure 2(G)). The FESEM image at 100,000× magnification of SYF revealed that the liposomes were also in nanosize (about 30 nm), spherical shape with smooth surface and homogenous distribution (Figure 2(H)).

#### Cryo-TEM study

Cryo-TEM study was performed to visualize the internal structure of NLs. Nanosize unilamellar vesicles were found in the Cryo-TEM images for both the types of NLs (Figure 2(I,J)). MGF had smaller size lyophilized vesicles than the size of SYF vesicles.

### *In vitro* investigations

#### *In vitro* drug release study

*In vitro* drug release study was conducted for both the formulations (MGF and SYF) to compare *in vitro* drug release patterns of both the formulations in two different release media. The data were plotted as percentage of cumulative drug release against time measured in hour (h) (Figure 2(K,L)). From the results, it appeared that cumulative 73.43% and cumulative 82.21% of AZT released from MGF in phosphate buffer media and in 50% serum, respectively, whereas cumulative 82.48% (phosphate buffer as media) and cumulative 84.51% (50% serum as media) of AZT released from SYF over a period of 24 h. Drug release was to an extent slower from MGF than SYF. However, a steady and sustained drug release pattern was observed for both the formulations.

#### Drug release kinetics analysis

To understand the drug release kinetic patterns, data were plotted using various kinetic equations and the corresponding correlation coefficients (represented here as *R*^2^ values) were determined (Supplementary Table 2). Data gave good linearity in Korsmeyer–Peppas’s kinetic model for both the formulations [*R*^2^ = 0.9742 (phosphate buffer as drug release media) and *R*^2^ = 0.9780 (50% serum as drug release media) for MGF and *R*^2^ = 0.9752 (phosphate buffer as drug release media) and *R*^2^ = 0.9751 (50% serum as drug release media) for SYF] as compared to the other kinetic models tested. The release exponent values (*n*) were 0.8387 and 0.8561 for MGF and SYF in phosphate buffer drug release media where as in 50% serum ‘*n*’ values were 0.7539 for MGF and 0.8179 for SYF, respectively. The ‘*n*’ values indicated non-Fickian diffusion pattern of release of drug from NLs in both types of drug release media.

#### *In vitro* cellular uptake study

**Figure 3.**
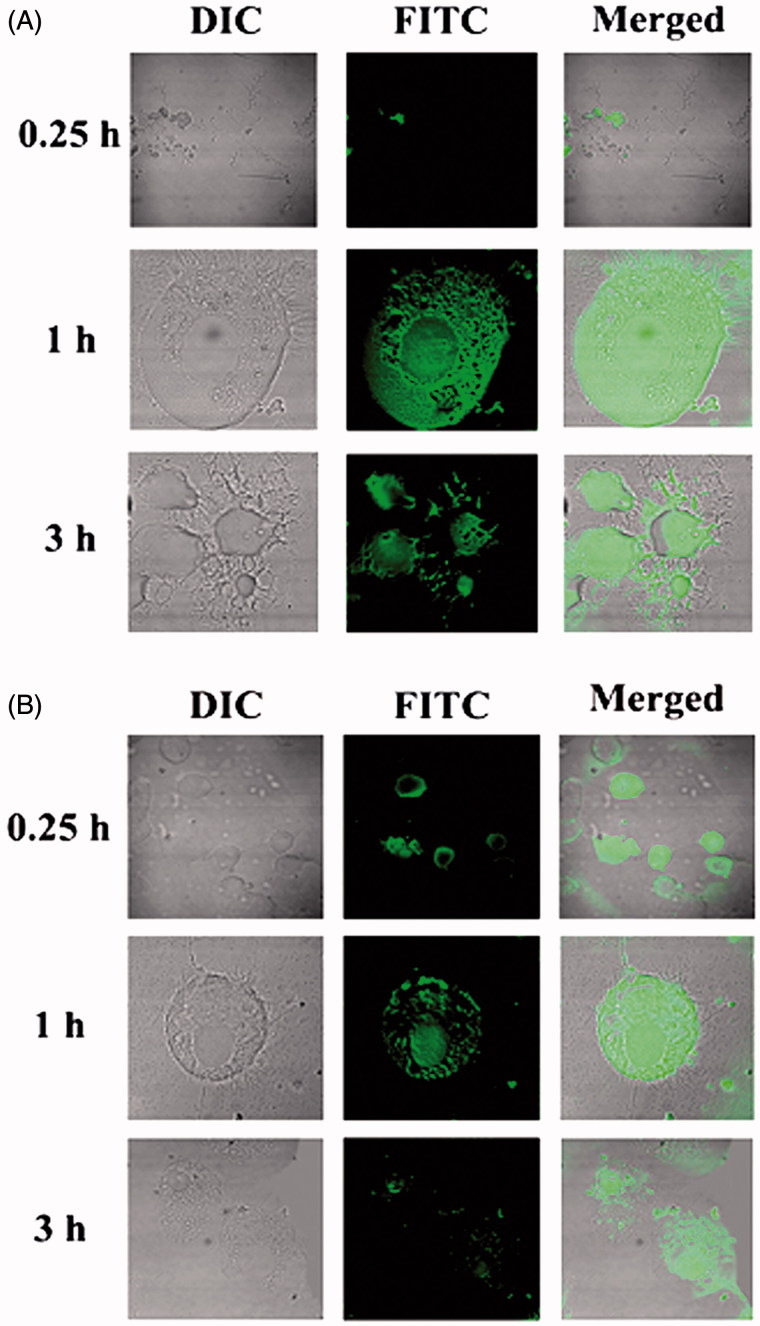
Investigation of *in vitro* cellular uptake of MGF and SYF by confocal microscopy. (A) *In vitro* cellular uptake study of fluorescein isothiocyanate labeled MGF (FITC-MGF) in U-87 MG human glioblastoma cells at various time points (0.25 h, 1 h, 3 h). (B) *In vitro* cellular uptake study of fluorescein isothiocyanate labeled SYF (FITC-SYF) in U-87 MG human glioblastoma cells at various time points (0.25 h, 1 h, 3 h). DIC: differential interference contrast images of U-87 MG human glioblastoma cells.

*In vitro* cellular uptake study of NLs was conducted in U-87 MG human glioblastoma cells by using fluorescent NLs to inspect the uptake ability of NLs in brain cells using confocal microscopy. From the results, it revealed that both the types of fluorescent NLs (FITC-MGF, FITC-SYF) were internalized by the cells (Figure 3(A,B)) and were localized in cytoplasm as well as nucleus. Cellular uptake of FITC-MGF increased with time till 3 h of the study (Figure 3(A)). However, for FITC-SYF, the cellular uptake was more in 1 h than 0.25 h and then decreased at 3 h (total duration of the study) (Figure 3(B)). By both confocal and conventional fluorescence microscopies, FITC treated cells were shown to internalize FITC in a time-dependent manner (Cole et al., 1990). Thus, addition of FITC in media alone as a control sample to treat cells would provide results which can mislead the actual findings of the FITC-labeled formulations. Hence, no FITC control was run.

### Stability study

FTIR spectra of the stored NLs (MGF, SYF) were compared with those of the freshly prepared formulations. No distinguish changes in spectrum were observed for the formulations stored at 4 °C. However, NLs stored at 25 °C showed the deformation of structure (data not shown), although drug assay results did not significantly vary the quantity of drug loaded in the formulation. Further, FTIR, DSC (Supplementary Figure 1), UV–visible spectroscopy, LC–MS/MS analysis of free drug, encapsulated drug and drug released showed that drug remained in the active form after encapsulation and released, and in the same active form.

### *In vivo* analysis

#### Gamma scintigraphy

**Figure 4.**
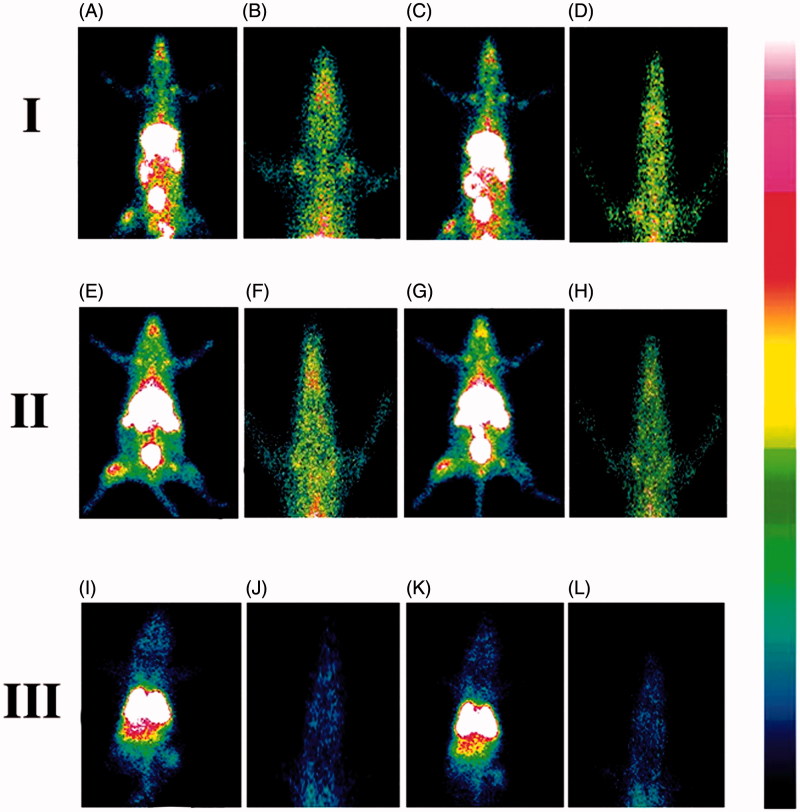
Gamma scintigraphy images of rats after receiving radiolabeled MGF/radiolabeled SYF/radiolabeled AZT. (I) rats received ^99m^Tc labeled MGF at 1 h (A, B) and at 5 h (C, D) post i.v. injection; (II) rats received ^99m^Tc labeled SYF at 1 h (E, F) and at 5 h (G, H) post i.v. injection; (III) rats received ^99m^Tc labeled free drug at 1 h (I, J) and at 5 h (K, L) post i.v. injection. A, C, E, G, I, and K are whole animal image; B, D, F, H, J, and L are magnified brain part.

The gamma scintigraphy was performed to investigate the ability of the NLs to cross BBB after the administration of ^99m^Tc labeled MGF, ^99m^Tc labeled SYF and ^99m^Tc labeled free drug (AZT) in different groups of Sprague-Dawley rats, respectively. The radioactivity signals were observed (Figure 4) and assessed in brain as well as in different organs (Supplementary Table 3) of the experimental animals which received radiolabeled NLs/AZT. The study showed that NLs were able to cross BBB and reached in brain. In animals treated with radiolabeled MGF, the intensity of signals in brain tissue was higher at 1 h as compared to 5 h (Figure 4(I)). The signals were stronger in brain at 1 h than 5 h in the animals received radiolabeled SYF (Figure 4(II)). At 5 h, the signals were stronger in the brain tissue of animals treated with radiolabeled MGF compared to radiolabeled SYF. In ^99m^Tc labeled free drug (AZT)-treated animals, very weak signal was noticed at 1 h and 5 h in brain tissue (Figure 4(III)) which indicated poor permeation of free AZT through BBB.

#### Biodistribution study

Biodistribution studies were conducted by administering ^99m^Tc labeled MGF, ^99m^Tc labeled SYF, and ^99m^Tc labeled free drug (AZT) in Sprague-Dawley rats through i.v. route at 1 h and 5 h after injection. In different organs including brain, accumulation of radiolabeled NLs as well as free drug was measured and data were reported as percentage of injected dose per gram (% ID per g) of tissue or organ (Supplementary Table 3). The values of residence time of radiolabeled formulation MGF/SYF in blood were 2.90-fold, 3.09-fold higher at 1 h and 4.08-fold, 3.94-fold greater than that of radiolabeled AZT at 5 h, respectively. On the other hand, kidney accumulation was distinctively more in free drug than NLs at 1 h as well as 5 h. Further, at 1 h, kidney accumulation of radiolabeled SYF was 2.75 time less than MGF but at 5 h this was 31.91% higher as compared to MGF. Enhancement of brain uptake values (by 18 folds and 19 folds, respectively, at 1 h and 36 times and 23 times, respectively, at 5 h, for MGF and SYF) was observed as compared to the brain uptake values of labeled AZT at those respective time points. Although brain uptake of ^99m^Tc labeled MGF was slightly less in values than those of SYF at 1 h but it was 56.52% higher compared to SYF at 5 h (Supplementary Table 3). For this reason, brain/blood ratio was 0.48 for MGF at 5 h which was greater than the value at 1 h whereas it was 0.32 for SYF at 5 h. Brain/blood ratio was predominantly higher for NLs compared to free AZT (Supplementary Table 3).

#### *In vivo* plasma pharmacokinetic study

**Figure 5.**
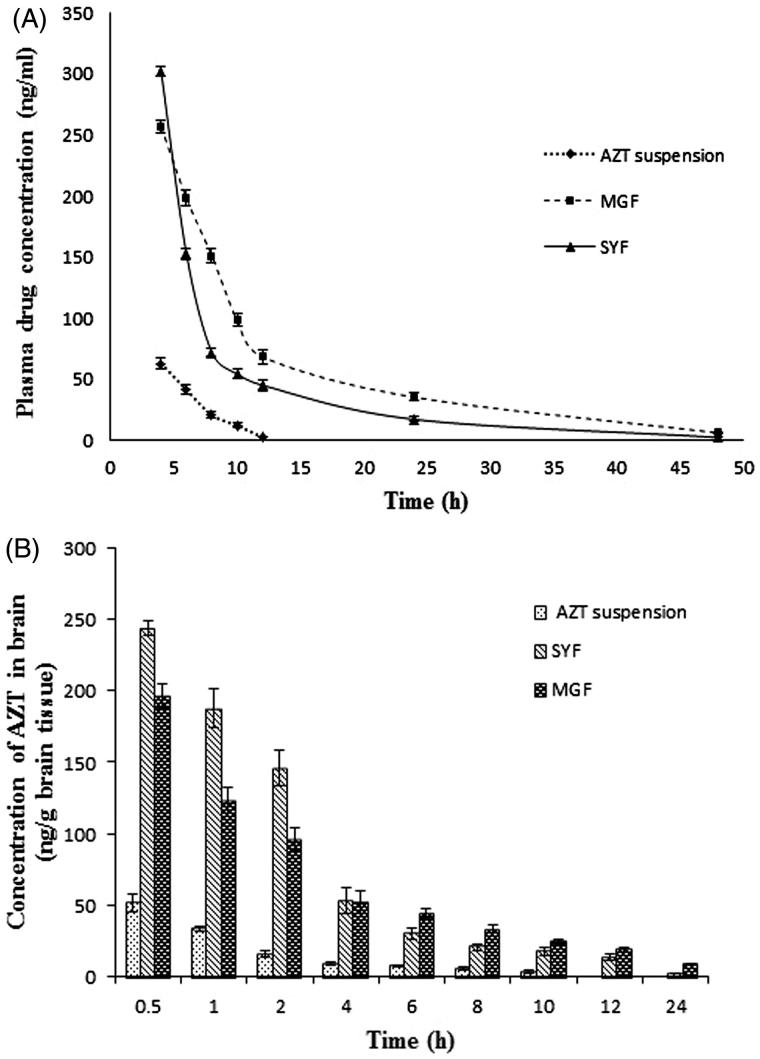
*In vivo* pharmacokinetic parameters in plasma and brain. (A) Plasma concentration of AZT–time profiles after i.v. administration of MGF/SYF/free drug (AZT) suspension in rats. (B) Concentration of AZT in brain after i.v. administration of MGF/SYF/AZT suspension in rats represented by bar diagram. Data showed mean ± SD (*n* = 3). SD of each point was represented by error bar. SD: standard deviation represented by deviation bar.

The plasma pharmacokinetic study was performed to observe the changes of pharmacokinetic parameters in Sprague-Dawley rats after i.v. administration of MGF/SYF/free AZT suspension. The mean plasma concentration of drug from MGF/SYF was higher than that of AZT in animals which had received AZT suspension, at the different experimental time points. The plasma concentration of AZT from MGF was comparatively higher than that of SYF after 4 h (Figure 5(A)). AUC_0–_*_t_* value for MGF was reasonably greater than those of SYF as well as free drug suspension (Table 1). AUMC_0–_*_t_* values of drug in rats received MGF/SYF were 13.76-fold/8.25-fold respectively greater than AUMC_0–_*_t_* value as detected in animals received free drug suspension. Half-life (*t*_1/2_) value of AZT from MGF was 8.44-fold higher than that of free AZT suspension and 1.27-fold higher than that of SYF, suggesting a predominantly sustained drug release from MGF. MRT values were 5.10-fold and 3.25-fold higher for MGF and SYF respectively than the MRT value found in rats treated with AZT suspension. This suggests maximally prolonged blood residence time of MGF among the experimental formulations. A predominant variation in *V*_ss_ values was also noticed for MGF (about two times greater than the value obtained from rat treated with AZT suspension and 1.5 times greater than the value obtained from rat treated with SYF). There was 63.21% decreased clearance of AZT from MGF as compared to free AZT suspension. Data revealed that MGF provided most favorable pharmacokinetic profile of AZT as compared to SYF and free AZT suspension.

**Table 1.** Plasma and brain pharmacokinetic parameters of AZT after i.v. administration of MGF/SYF/free drug (AZT) suspension in rats.

Pharmacokinetic parameters	AZT suspension	MGF	SYF
Plasma pharmacokinetic profile			
*t*_1/2_ (h)	1.15 ± 0.04	9.71 ± 0.21^a^^,^^b^	7.64 ± 0.08^a^
AUC_0–_*_t_* (h ng ml^–1^)	2003.27 ± 24.63	5405.57 ± 151.03^a^	5107.23 ± 153.91^a^
AUMC_0–_*_t_* (h^2^ ng ml^–1^)	2529.70 ± 188.70	34806.50 ± 2475.71^a^^,^^b^	20881.90 ± 1198.92^a^
Cl (L h^–1^ kg^–1^)	2.99 ± 0.04	1.10 ± 0.03^a^	1.17 ± 0.03^a^
MRT (h)	1.26 ± 0.08	6.43 ± 0.28^a^^,^^b^	4.09 ± 0.24^a^
*V*_ss_ (L kg^–1^)	3.83 ± 0.19	7.88 ± 0.19^a^^,^^b^	5.03 ± 0.38^a^
Brain pharmacokinetic profile			
AUC_0–_*_t_* (h ng ml^–1^)	141.17 ± 12.08	888.23 ± 28.67^a^	875.73 ± 53.86^a^
AUMC_0–_*_t_* (h^2^ ng ml^–1^)	386.53 ± 35.11	5396.33 ± 236.87^a^^,^^b^	3574.03 ± 290.51^a^
MRT (h)	2.73 ± 0.06	6.07 ± 0.15^a^^,^^b^	4.07 ± 0.06^a^
Cl (L h^–1^ kg^–1^)	37.33 ± 4.66	5.9 ± 0.17^a^	6.73 ± 0.35^a^

*t*_1/2_: half-life; AUC_0–_*_t_*: area under the concentration–time curve from time of injection (*t* = 0) to a determined time point; AUMC_0–_*_t_*: area under the first moment curve; Cl: clearance; MRT: mean residence time; *V*_ss_: steady state volume of distribution.

*Note*: Data were expressed as mean ± SD of three separate observations.

a Data were significantly different (*p* < .05) where MGF and SYF were compared with AZT suspension. It was assessed by one-way analysis of variance (ANOVA) through Tukey–Kramer’s multiple comparisons test.

b Data were significantly different (*p* < .05) where MGF was compared with SYF. It was assessed by one-way analysis of variance (ANOVA) through Tukey–Kramer’s multiple comparisons test.

#### *In vivo* brain pharmacokinetic study

Till 24 h (the total duration of the study), AZT level was detectable in brain from MGF/SYF (Figure 5(B)). However, AZT could not be detected in the brain of rats treated with AZT suspension after 10 h of the study. AZT level in brain was found to be maximum from SYF followed by MGF in 0.5 h. The values were five- and four-fold higher respectively as compared to the values detected in animals treated with AZT suspension at 0.5 h. AZT level was 25% higher in the brain of rats treated with SYF as compared to MGF at 0.5 h. With the increasing duration of time, AZT levels were decreasing in all the cases. Interestingly, at 4 h of the investigation the values of AZT concentration in brain were almost same in rats treated with SYF/MGF. However, from then on, till 24 h of the study, AZT levels in brain were found to be more (46.76% more in value at 6 h, 263.75% more in value at 24 h) in rats treated with MGF as compared to those treated with SYF. Treatment of AZT through MGF enhanced AUMC_0–_*_t_* value by 50.99% and MRT value by 49.14% as compared to the rats treated with SYF. The data were also substantiated by clearance values (Table 1).

## Discussion

The present investigation was intended to understand the capability of ML to act as base material of lipid nanocarrier to deliver water soluble drug through BBB to brain. Continuous tight junctions of endothelial cells of brain capillaries and several transmembrane proteins seal the paracellular pathways and effectively block the free diffusion of polar solutes (such as AZT) from blood along these potential paracellular pathways, causing denial of access to brain interstitial fluid (Ballabh et al., 2004; Abbott, 2005). Blood–brain barrier predominantly impedes entry from blood to brain of virtually all molecules, other than those that are small and lipophilic (e.g. nanoliposome) or those (such as essential nutrients, precursors and cofactors) that enter the brain through active transport mechanism (Alavijeh et al., 2005; Agrawal et al., 2017). Thus, lipophilicity and size of nanoliposome tend to lead higher permeation across BBB as compared to the polar drug or comparatively polar phospholipid vesicles. Since AZT is a highly water soluble drug that does not cross BBB efficiently (Rautio et al., 2008; Weiss et al., 2009) and the nanosize lipid vesicles have been shown to permeate through BBB effectively (Masserini, 2013; Vieira & Gamarra, 2016; Li et al., 2017), we developed two types of NLs (MGF and SYF) applying lipid layer hydration technique by using ML/SL. A comparative physicochemical and biopharmaceutical analysis on those two types of formulations were also performed.

FTIR data analysis revealed that there was no chemical interaction between the drug and the excipients. However, there were some physical interactions between the drug and excipients and between the molecules of excipients. Such physical interactions might have a role to form the spherical nanostructure of the drug carrier (Rudra et al., 2010) and to retain the drug in the lipid layer, causing slower diffusion of drug through the membrane (Rudra et al., 2010). Again, absence of peak of AZT in the lyophilized ML-based NLs was due to the entire encapsulation of AZT in the formulation. Presence of peak of AZT in SYF suggests the availability of free drug on the surface of the formulation.

Various process parameters such as temperature and speed of hydration, duration of sonication, time and speed of centrifugation, duration of freeze drying and ratio of the constituents were optimized to develop formulations with maximum drug loading, homogeneous and uniform particle size, desirable zeta potential value and smooth surface structure, among the experimental formulations. Out of the various experimental formulations, the best optimized formulation of each category (MGF/SYF) was selected and reported here.

Lower drug loading was observed in MGF as compared to SYF. AZT is a highly water soluble drug (Singh et al., 2010; Nath et al., 2011). SYF had hydrophobic phospholipid and CHO. Presence of phosphate group in SL makes it comparatively more hydrophilic than ML (Jones, 2008). Presence of SA, OA, and PA possibly caused comparatively less partitioning of drug into the lipid layer causing less drug loading in MGF. Yield of SYF was nearly about 6% less as compared to MGF and the possible reason may be the recovery problem due to slightly stickiness of SYF for presence of SL (Das et al., 2015).

Nanosize SYF had larger size (38.49% larger) than MGF. This could be because of the presence of phospholipid in SYF. Phospho moiety owing to the hydrophilic nature of the phosphate group (Jones, 2008) might have been pulled with more tension by water molecules toward the bulk of the liquid during the formation of the liposomal structure, resulting in comparatively larger size. PDI values of both the formulations were nearly similar. PDI value is a very crucial indicator for size distribution, stability, and uniformity of NLs (Masarudin et al., 2015). Lower PDI value signifies more monodisperse pattern with better stability of NLs. On the other hand, higher PDI value indicates aggregation of particles with low stability (Masarudin et al., 2015). The PDI value 0.1–0.25 is desirable for uniform distribution but the value more than 0.5 is an indication of poor homogeneity (Gharib et al., 2014). In our study, both the formulations were mostly uniform in size and homogeneously distributed. Zeta potential is also considered as a parameter for confirmation of physical stability (Dey et al., 2016; Shaw et al., 2017). If electric charge of NLs surface is high, then zeta potential of NLs will also show high value. This means strong repellent forces among the vesicles of NLs are able to inhibit agglomeration of NLs in suspension. Normally, zeta potential value (more than +30 mV or less than –30 mV) indicates good stability of NLs in colloidal dispersion (Dey et al., 2016). In our study, nearly similar zeta potential values (about –70 mV) were achieved for MGF and SYF. This denotes that both the formulations had prolonged and better physical stability in colloidal suspension. The negatively charged NLs are removed slowly than the positive one, which suggests longer blood residence time of negatively charged drug carriers (Satapathy et al., 2016). Thus, both the experimental formulations are expected to possess extended blood residence time.

FESEM provides information related to 3D structure as well as surface property (Tripathi et al., 2017). FESEM study reveals that both the formulations had spherical structure with smooth surface and they were homogeneously distributed with nonappearance of any agglomerate.

The Cryo-TEM images show that MGF/SYF had unilamellar spherical nanosize structure with an intact lamellarity. Lipid layer was present at the external side and aqueous part was enclosed by the lipid layer. Dark spot in the inner aqueous core and dark outer layer revealed the presence of hydrophilic drug in a suspended condition in the aqueous core as well as in the outer lipid layer.

Drug was found to release in a slower and more sustained manner from MGF compared to SYF in both types of drug release media. This could be due to the lipid composition of the NLs. SYF fundamentally consisted of phospholipid which contains polar phosphoryl and basic groups and they make the phospholipid more hydrophilic (Jones, 2008) than the ML which does not contain any phospholipid. Since AZT is a highly water soluble drug (Singh et al., 2010; Nath et al., 2011), it seems to permeate more through phospholipid than the ML.

The drug release pattern from both the NLs in phosphate buffer media as well as 50% serum was best fitted (based on *R*^2^ value) with Korsmeyer–Peppas’s kinetic model. Release component (*n*) value suggests for an anomalous non-Fickian diffusion pattern of drug molecules (Pattnaik et al., 2012). Further, Korsmeyer–Peppas’s kinetic model suggests that AZT release from the experimental NLs followed diffusion and erosion process (Pattnaik et al., 2012).

Cellular uptake of both the NLs in U-87MG human glioblastoma cells showed that NLs were localized in the cytoplasm and in the nucleus after internalization by the cells. But FITC-MGF was intense in cells in a time dependent manner whereas FITC-SYF concentration in cells initially increased (at 1 h as investigated) and then decreased with the time (at 3 h, total duration of the investigation). Possibly phospholipid vesicles were metabolized faster than MGF by glioma cells (Carnielli et al., 1998; Klein, 2002).

At 4 °C, absence of changes in spectra for formulations indicates that there was no physico-chemical reaction occurred between the drug and the excipients during storage condition. So, drug remains as a stable form in stored formulation during storage condition at 4 °C. At 25 °C, although FTIR spectra did not significantly vary for the experimental formulations, the deformity of structures of NLs forced us to reject the consideration of 25 °C to store the formulation. However, drug assay showed that the drug was stable in the formulation at 25 °C.

We performed gamma scintigraphic investigation and brain pharmacokinetic study to compare the capability of MGF and SYF to cross BBB. Gamma scintigraphic images gave clear picture of localization of radiolabeled NLs/radiolableled AZT in whole body of the experimental rats. From the gamma scintigraphic images, it revealed that both the radiolableled NLs were capable to cross BBB and reached in brain. The intensity of signal in brain was stronger at 1 h as compared to 5 h for both MGF and SYF (as observed from signal intensity bar given with the photograph). At 5 h, the signal intensity of MGF in brain was more pronounced than that of SYF, suggesting longer retention of radiolabeled MGF than radiolabeled SYF in brain after crossing BBB. Very weak signal at 1 h and 5 h for ^99m^Tc labeled free drug (AZT) signifies poor ability of AZT to cross BBB. Both the NLs successfully crossed BBB possibly due to their nano-vesicular structure (Li et al., 2017) and longer retention of MGF in brain compared to SYF might be due to the variable lipid characteristics of MGF than SYF which had phospholipid in its structure.

In biodistribution study, radiolabeled MGF obtained from brain/blood ratio maintained its level persistently as compared to radiolabeled SYF, suggesting MGF was able to provide sustaining drug level in brain and blood better than SYF. Application of MGF showed presence of more amount of drug in brain with the increasing duration of the experiment as compared to that of SYF owing to an increased presence of MGF in brain. The presence of more MGF in brain/blood ratio as compared to SYF might be due to the sustained drug release and less clearance of drug through liver and kidney as compared to those of SYF. Predominant hepatic clearance of free AZT is also noteworthy to mention. The investigation reported that it is possible for a compound to possess a long half-life in blood, but a short half-life in brain or even not to permeate BBB at all (Dawson et al., 2001). Hence, if a drug or formulation remains longer time in blood, it does not necessarily provide enhanced brain level of the drug (Dawson et al., 2001; Vieira & Gamarra, 2016). On the other hand, almost all things injected in blood must go into liver and liver does not have any barrier like BBB. Hence, the access of drug/formulation takes place much faster with a greater amount in liver than brain. Thus, MGF can tackle the short elimination half-life, low bioavailability, frequent dosing and dose-dependent toxicity of AZT (Mandal & Tenjarla, 1996; Blum et al., 1988; Oh et al., 1998; Thomas & Panchagnula, 2003) more effectively than SYF.

After i.v. administration, at 4 h, SYF showed more drug level in blood as compared to MGF. It dropped sharply with the increasing duration till 8 h which indicates rapid distribution of SYF compared to MGF. However, MGF showed slow distribution and significantly (*p* < .05) longer blood residence time as compared to SYF which also increased *t*_1/2_ value of AZT. AUC_0–48_ and AUMC_0–48_ were significantly more for MGF. It could be due to the longer MRT and less renal clearance of MGF compared to SYF. Reports suggest that longer MRT and less renal clearance increase AUMC and AUC (Dey et al., 2016; Satapathy et al., 2016).

Drug concentration in brain was initially 25% more for SYF than MGF. The drug level in brain from MGF/SYF eventually reduced with the duration. At 4 h, the drug level in brain from MGF/SYF was more or less same. Interestingly, brain drug concentration from SYF dropped sharply after 4 h whereas it dropped in a much slower manner for MGF till 24 h of the study where brain drug level was about 260% more than that obtained from SYF. One of the most important features of brain is that it is completely separated from blood by BBB. In our study, blood perfusion was done before collection of brain from the animals to estimate drug in brain. Blood perfusion precludes the presence of drug in brain vasculatures and rather suggests its accumulation in brain, as drug has to cross BBB to reach brain endothelial cells (Ballabh et al., 2004; Abbott, 2005; Alavijeh et al., 2005). Thus, *in situ* perfusion provides a measure of brain uptake and the brain/plasma ratio of the drug (as done in the present study) provides a partial measure (Takasato et al., 1984; Abbott, 2005; Alavijeh et al., 2005). Since both the studies have been done, it supports that drug reached in brain. It is also an indicative of cross of the drug-loaded formulations through BBB. Sustained drug release, less clearance, and enhanced MRT of MGF in brain could be responsible for such enhancement. These further reflected in the enhanced availability of drug in brain from MGF compared to SYF. Lipid composition of MGF might be responsible for such variation.

## Conclusions

In the study, nanosize drug delivery systems were developed successfully utilizing ML/SL to deliver AZT in brain across BBB. Both the formulations had sustained drug release profiles. *In vivo* gamma scintigraphy study and brain pharmacokinetic investigation revealed that both the formulations sufficiently reached brain through BBB, but MGF had better pharmacokinetic profile than that of SYF with respect to sustained drug release, prolonged blood residence time as well as brain residence time and increased *t*_1/2_. Thus, ML may be utilized as a new and effective carrier material to deliver AZT effectively in brain. ML could be an emerging pharmaceutical for developing various therapeutic strategies to deliver drugs to brain and other organs. However, further studies are warranted in the area.

## Supplementary Material

IDRD_Mukherjee_et_al_Supplemental_Content.zip
