# Supplementary figures and images for: Lipid-based nanocarrier efficiently delivers highly water soluble drug across the blood–brain barrier into brain
Source: Drug Deliv. 2018 Feb 9;25(1):504–16. doi: 10.1080/10717544.2018.1435749 (PMC6058568; doi:10.1080/10717544.2018.1435749)

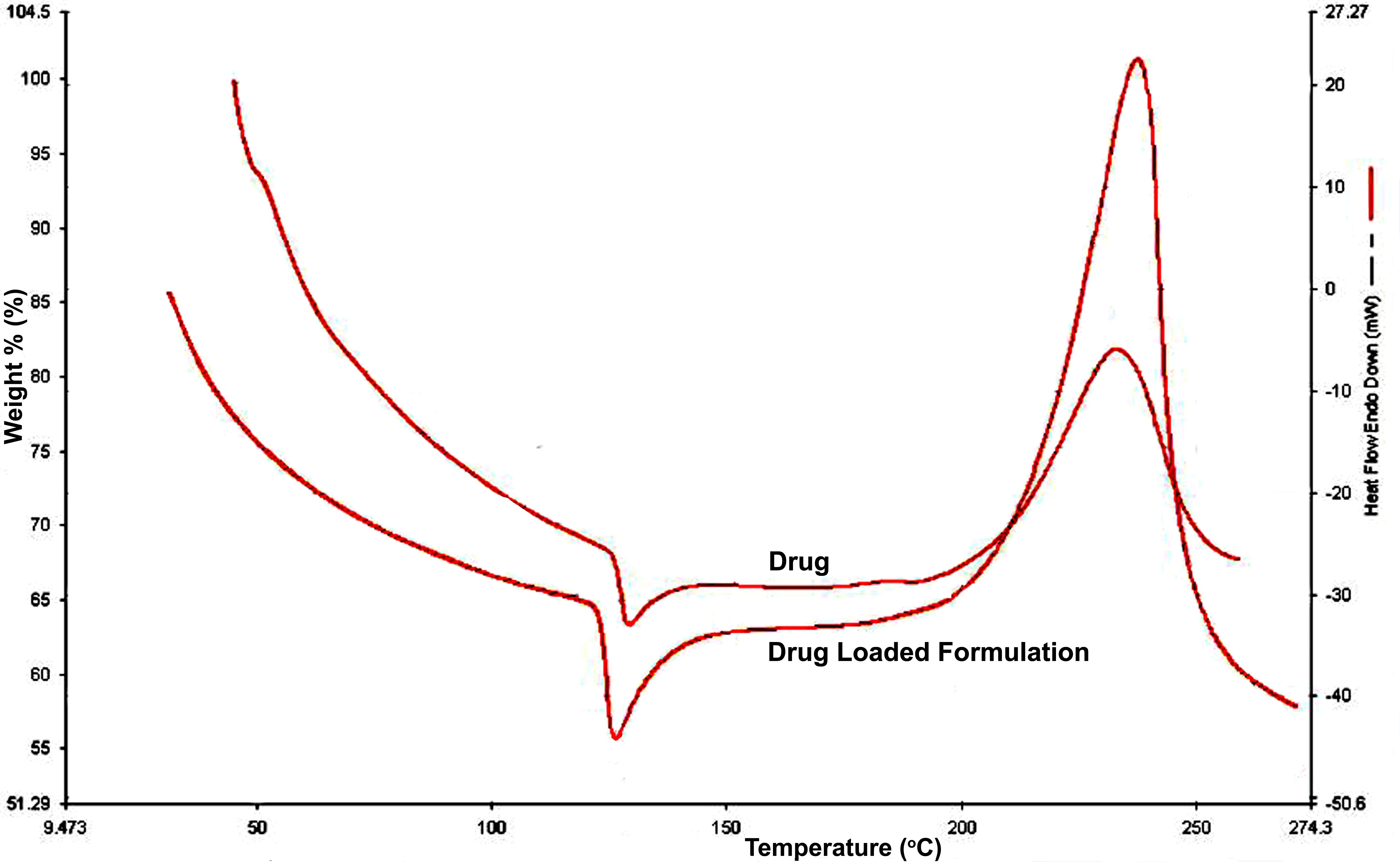

Supplement: IDRD_Mukherjee_et_al_Supplemental_Content.zip [file IDRD_A_1435749_SM8013.zip › Supplementary Figure 1.tif]
