# Supplementary material for: Lipid-based nanocarrier efficiently delivers highly water soluble drug across the blood–brain barrier into brain
Source: Drug Deliv. 2018 Feb 9;25(1):504–16. doi: 10.1080/10717544.2018.1435749 (PMC6058568; doi:10.1080/10717544.2018.1435749)
Supplement: IDRD_Mukherjee_et_al_Supplemental_Content.zip [file IDRD_A_1435749_SM8013.zip › Legends to the supplementary figure.doc]

**Legend to the Figure**

Supplementary Figure 1. Differential scanning calorimetry thermograms of drug (zidovudine) and drug loaded formulation (MGF), showing that the drug has a sharp endothermic peak that corresponds to melting in the range.124-125ºC.
