# Supplementary material for: Lipid-based nanocarrier efficiently delivers highly water soluble drug across the blood–brain barrier into brain
Source: Drug Deliv. 2018 Feb 9;25(1):504–16. doi: 10.1080/10717544.2018.1435749 (PMC6058568; doi:10.1080/10717544.2018.1435749)
Supplement: IDRD_Mukherjee_et_al_Supplemental_Content.zip [file IDRD_A_1435749_SM8013.zip › Supplementary Table 1.doc]

**Supplementary Table 1. Composition, % yield and % drug loading of selected experimental formulations^b^.**

| Formulation code | Composition | Ratio  (w/w) | % yield^a^ | Practical % drug loading^a^ |
| --- | --- | --- | --- | --- |
| MGF | CHO, ML, AZT | 5: 15: 3 | 60.87 ± 5.92 | 5.7 ± 0.37 |
| SYF | CHO, SL, AZT | 5: 15: 3 | 54.34 ± 4.79 | 7.0 ± 0.23 |

Note: ^a^ Data were expressed as mean ± SD (n = 3). ^b^ MGF: Lipid (ML) based formulation, SYF: Soya lecithin (SL) based formulation. SD,standard deviation.
