# Supplementary material for: Lipid-based nanocarrier efficiently delivers highly water soluble drug across the blood–brain barrier into brain
Source: Drug Deliv. 2018 Feb 9;25(1):504–16. doi: 10.1080/10717544.2018.1435749 (PMC6058568; doi:10.1080/10717544.2018.1435749)
Supplement: IDRD_Mukherjee_et_al_Supplemental_Content.zip [file IDRD_A_1435749_SM8013.zip › Supplementary Table 2 (1).doc]

Supplementary Table 2. *In vitro* drug release kinetics of MGF and SYF with R^2^ and “n” values.

| Phosphate buffer using as drug release media | | |
| --- | --- | --- |
| Kinetic model | MGF | SYF |
|  | Kinetic equation | Kinetic equation |
| Zero order kinetics | y = 3.3472x + 13.839 R² = 0.8080 | y = 3.8137x + 15.178 R² = 0.8018 |
| First order kinetics | y = -0.0272x + 1.9585 R² = 0.8871 | y = -0.0361x + 1.969 R² = 0.8968 |
| Higuchi kinetics | y = 19.829x - 11.022 R² = 0.9371 | y = 22.617x - 13.209 R² = 0.9318 |
| Hixson-Crowell kinetics | y = -0.0777x + 4.4597 R² = 0.8634 | y = -0.0972x + 4.464 R² = 0.8697 |
| Koresmeyer-Peppas kinetics | y = 0.8387x + 0.8849 R² = 0.9742  n = 0.8387 | y = 0.8561x + 0.9188 R² = 0.9752  n = 0.8561 |
| 50 % serum using as drug release media | | |
| Kinetic model | MGF | SYF |
|  | Kinetic equation | Kinetic equation |
| Zero order kinetics | y = 3.7114x + 17.159 R² = 0.7958 | y = 3.9148x + 16.204  R² = 0.7911 |
| First order kinetics | y = -0.0356x + 1.9562 R² = 0.8874 | y = -0.039x + 1.9681 R² = 0.8875 |
| Higuchi kinetics | y = 22.056x - 10.579 R² = 0.9287 | y = 23.248x - 13.014 R² = 0.9219 |
| Hixson-Crowell kinetics | y = -0.0955x + 4.4248 R² = 0.8611 | y = -0.103x + 4.4521 R² = 0.8594 |
| Koresmeyer-Peppas kinetics | y = 0.7539x + 1.0228 R² = 0.9780  n = 0.7539 | y = 0.8179x + 0.9696 R² = 0.9751  n = 0.8179 |

Note: R^2^, correlation coefficient; n, release exponent.
