# Supplementary material for: Lipid-based nanocarrier efficiently delivers highly water soluble drug across the blood–brain barrier into brain
Source: Drug Deliv. 2018 Feb 9;25(1):504–16. doi: 10.1080/10717544.2018.1435749 (PMC6058568; doi:10.1080/10717544.2018.1435749)
Supplement: IDRD_Mukherjee_et_al_Supplemental_Content.zip [file IDRD_A_1435749_SM8013.zip › Supplementary Table 3.doc]

**Supplementary Table 3. Biodistribution studies of ^99m^Tc labeled MGF / SYF and ^99m^Tc labeled free drug (AZT) in rats at 1 h and 5 h post i.v. injection time period.**

| Organ | MGF | | SYF | | AZT | |
| --- | --- | --- | --- | --- | --- | --- |
|  | 1h | 5h | 1h | 5h | 1h | 5h |
| Heart | 0.06 ± 0.02 | 0.18 ± 0.04 | 0.05 ± 0.01 | 0.14 ± 0.03 | 0.31 ± 0.01 | 0.15 ± 0.03 |
| Blood | 1.97 ± 0.18 | 1.51 ± 0.15 | 2.10 ± 0.21 | 1.46 ± 0.11 | 0.68 ± 0.19 | 0.37 ± 0.13 |
| Liver | 27.61 ± 2.78 | 36.25 ± 2.43 | 25.22 ± 2.34 | 38.21 ± 2.31 | 32.28 ± 3.21 | 38.23 ± 3.11 |
| Lung | 2.93 ± 1.33 | 1.63 ± 1.43 | 3.51 ± 1.26 | 2.63 ± 1.55 | 1.66 ± 1.36 | 2.15 ± 1.35 |
| Spleen | 2.65 ± 0.26 | 1.51 ± 0.19 | 3.99 ± 0.19 | 1.91 ± 0.21 | 1.53 ± 0.24 | 2.23 ± 0.27 |
| Muscle | 0.06 ± 0.01 | 0.09 ± 0.01 | 0.03 ± 0.00 | 0.06 ± 0.01 | 0.15 ± 0.02 | 0.19 ± 0.05 |
| Intestine | 1.64 ± 0.29 | 2.07 ± 0.26 | 1.56 ± 0.31 | 2.96 ± 0.23 | 1.11 ± 0.28 | 2.23 ± 0.29 |
| Stomach | 0.68 ± 0.17 | 0.75 ± 0.13 | 0.38 ± 0.09 | 0.56 ± 0.07 | 0.45 ± 0.09 | 0.57 ± 0.11 |
| Kidney | 0.33 ± 0.11 | 0.47 ± 0.12 | 0.12 ± 0.05 | 0.62 ± 0.08 | 4.56 ± 0.19 | 6.45 ± 0.21 |
| Brain | 0.90 ± 0.16 | 0.72 ± 0.09 | 0.95 ± 0.21 | 0.46 ± 0.11 | 0.05 ± 0.01 | 0.02 ± 0.00 |
| Brain / Blood | 0.46 | 0.48 | 0.45 | 0.32 | 0.07 | 0.05 |

Note: Results are expressed in % mean of injected dose per gram of organ/tissue ± SD (n = 5).
